# Infected Osteoclastoma of the Knee: An Unusual Presentation

**DOI:** 10.1155/2014/948536

**Published:** 2014-03-04

**Authors:** O. B. Pattanashetty, B. B. Dayanand, Yogesh Mapari, Monish Bami

**Affiliations:** ^1^Department of Orthopaedics, Shri BM Patil Medical College, Bijapur, Karnataka 586103, India; ^2^Jivala, Plot No. 6, Welcome Nagar, Behind Janakpuri Colony, Garkheda Area, Aurangabad, Maharashtra 431005, India

## Abstract

*Introduction*. Giant cell tumor is a benign or locally aggressive tumor of uncertain origin that appears in mature bone, most commonly in the distal femur, proximal tibia which characteristically extends right up to the subarticular bone plate. *Case Report*. We report here a 35-year-old female presenting with swelling of the left knee. On examination, the swelling was solitary, about 20 × 15 cm in size with the skin over the swelling stretched and glistening. On the fifth day of hospital stay, the swelling burst open and blood tinged pus started pouring out. X-ray and MRI scan showed a well-defined T2 hyperintense expansile eccentrically located osteolytic lesion involving the metaphyseal region of the proximal tibia and extending into the subarticular space and multiple T1/T2 hypointense septations are noted within the lesion suggestive of osteoclastoma. The patient was counseled regarding the tumor and prognosis and various treatment options. She was treated successfully with above knee amputation. The tissue was sent for histopathology which confirmed osteoclastoma. *Conclusion*. It is important to recognize giant cell tumors early, so that they can be treated promptly with local measures to prevent morbidity and mortality in young adults.

## 1. Introduction

Giant cell tumor is so named because it contains profusion of multinucleated osteoclast-type giant cells, giving rise to the synonym osteoclastoma. The term osteoclastoma was first used in Great Britain by Stewart in 1922. It is a benign but locally aggressive neoplasm representing approximately 5% of all primary bone tumors. It usually arises in patients in their twenties to forties. The tumor is slightly more common in female. They are typically epiphyseometaphyseal tumors, with the majority involving the distal femur and proximal tibia. The clinical history of a giant cell tumor usually includes pain and limitation of motion because of the tumor's proximity to the joint space. Swelling occurs late in the course of the giant cell tumor. Patients presenting to the doctor early in the course of the tumor can be treated with local surgery such as thorough curettage and cryosurgery or burring of the cavity with or without phenol installation. The lesional area may then be packed with allograft or polymethyl methacrylate. Such treatment reduces the local recurrence rate to less than 20% and the prognosis is excellent if it does not recur locally [1].

If this tumor is not diagnosed and treated early, it continues to grow in size causing massive local tissue destruction and may get secondarily infected thereby requiring amputation.

## 2. Case Report

A 35-year-old female patient presented to our opd with swelling of the left knee for 6 years. Initially, the swelling was small, which gradually increased in size, covering the entire anterior aspect of the knee.

**Figure 1:**
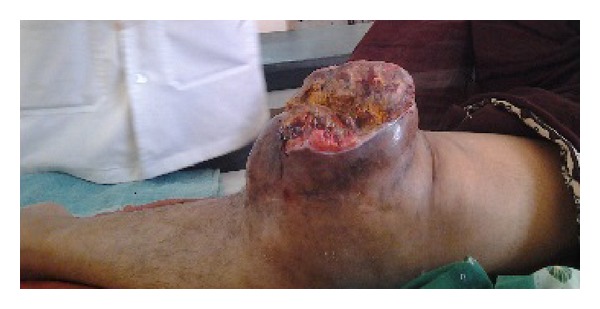
Ruptured tumor at the knee joint.

**Figure 2:**
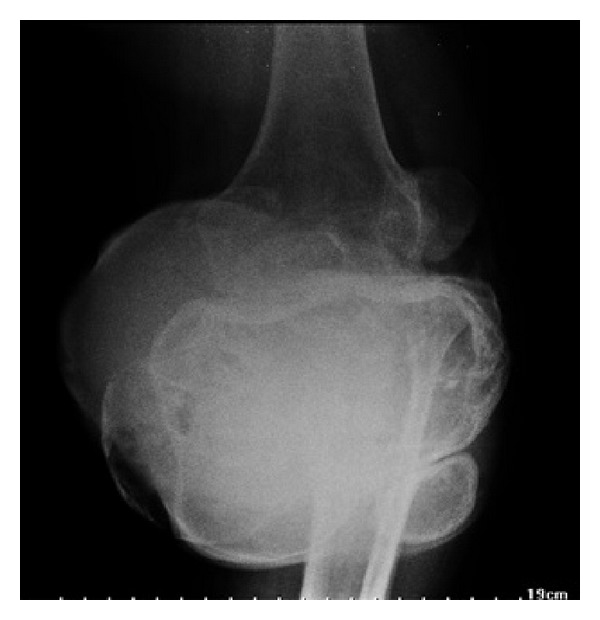
X-ray of the right knee showing osteolytic lesion in proximal tibia.

**Figure 3:**
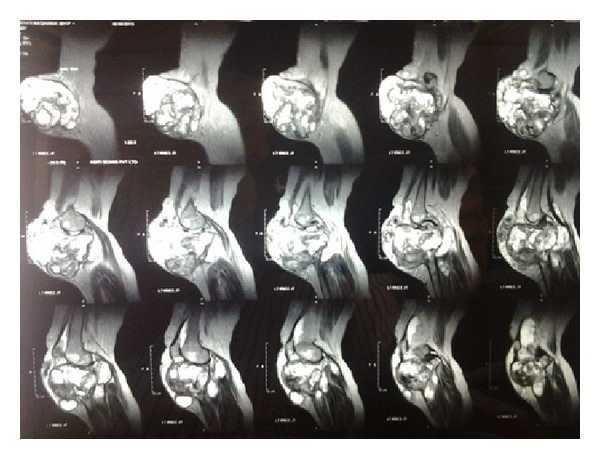
MRI of the right knee joint.

Also, she complained of pain and loss of movements of the knee with inability to walk. There was no history of discharge from the lesion, fever, weight loss, or other joint pains. General physical examination was normal except for pallor. Systemic examination was also normal. On local examination, the swelling was solitary, about 20 × 15 cm in size with the skin over the swelling stretched and glistening. On the fifth day of hospital stay, the swelling burst open and blood tinged pus started pouring out. The pus was sent for culture which showed *Staphylococcus aureus* which was sensitive to piperacillin and tazobactam which was started immediately. All routine blood investigations were carried out. The patient was noted to have severe anemia and her random blood sugar level was 385 mg%. Her serum calcium and alkaline phosphatase levels were within normal range. X-ray and MRI scan of left knee showed a well-defined T2 hyperintense expansile eccentrically located osteolytic lesion involving the metaphyseal region of the proximal tibia and extending into the subarticular space and multiple T1/T2 hypointense septations were noted within the lesion suggestive of osteoclastoma (Figure 2). Her hemoglobin was then improved by giving her serial packed red cells transfusions. She was not a known case of diabetes mellitus and her sugar was controlled by putting her on regular insulin subcutaneously as per sliding scale every 8 hours. Chest X-ray and bone scan were done to rule out any metastasis (Figure 3). The patient was counseled regarding the tumor and prognosis and various treatment options but due to financial constraints the patient was treated with above knee amputation (Figure 1). Sample from the lesion was sent for histopathological reporting which showed multiple multinucleated giant cells in a background of stromal cells, confirming it to be giant cell tumor. Elastic stockinette was wrapped over the postoperative dressing to prevent edema accumulation in the residual limb. Postoperatively, she was asked to lie supine or prone (to tolerance) to prevent hip flexion contracture. She was continued on sliding scale as mentioned before till the sugar levels returned to normal. Cardiovascular training was initiated as appropriate to patient tolerance in order to improve endurance and functional mobility tolerance. The patient started on bed mobilization and therapeutic exercises. Ambulation was started on the 5th day as tolerated with assistive device.

Distance of ambulation was progressed to patient's tolerance. Exercises were encouraged when the patient was on bed or sitting in chair. Dressings were done on the 3rd, 6th, and 9th postoperative days. Pain was controlled with adequate intravenous analgesics. Intravenous antibiotics were given for 4 days after which oral antibiotics were continued till suture removal which was done on the 12th postoperative day. There was no discharge or gaping at the stump site. Patient was then discharged on the 14th postoperative day after giving her oral hypoglycemic drugs. The patient returned for followup after 1 month and had no residual symptoms with completely healed stump (Figure 5). Ambulation activities with a prosthesis began during the 11th week after amputation after meticulous preprosthetic management.

## 3. Discussion

**Figure 4:**
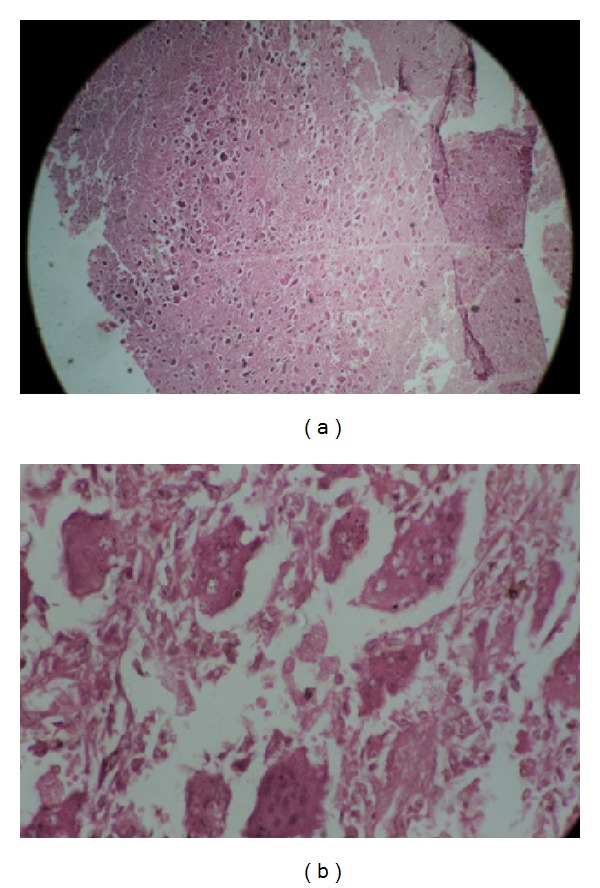
Histopathological slides showing giant cells suggestive of osteoclastoma.

**Figure 5:**
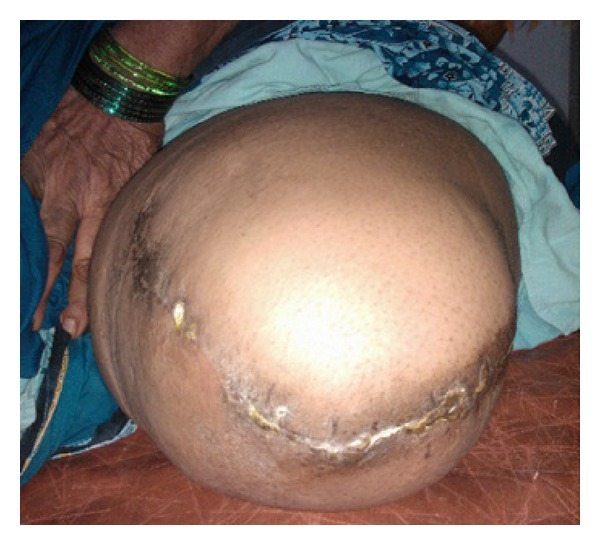
Healed stump after 1 month.

Giant cell tumors are benign but locally aggressive tumors and, at times, can be malignant. They can be well treated with local surgical measures if diagnosed early. Radiation therapy has been used for the treatment of giant cell tumor of bone since 1906 [2, 3]. Radiation therapy has been largely abandoned for giant cell tumors as this lesion has been labeled as radioresistant because of frequent tumor progression following primary or postoperative radiotherapy. Dahlin reported recurrence in 47% receiving irradiation following simple curettage, compared with 42% following curettage alone[3]. The failure of elective irradiation to improve control rates after incomplete excision has been noted elsewhere [4, 5]. Modern megavoltage irradiation remains a viable therapeutic modality for the giant cell tumor. Bennett et al. [6], reporting the University of Florida experience and reviewing the recent world literature, noted an overall local control of 77% following radiotherapy in 97 cases. The majority of these patients were irradiated primarily for gross tumor. Radiotherapy has been reported successful for giant cell tumor of the skull [4], spine [7–9], nonepiphyseal long bone locations, and metastatic deposits [4]. Radiotherapy is best reserved for those giant cell tumors not amenable to modern resection or curettage with aggressive chemical installation. Vascular space invasion, tumor ploidy, symptom duration, and histopathologic grade have not been found to reliably predict clinical course in surgical series of giant cell tumors [10, 11]. Recurrent giant cell tumors may demonstrate malignant osteoclastoma or frank sarcomatous change to fibrosarcoma or osteosarcoma (Figure 4). Benign giant cell tumors have been noted to metastasize to the lungs. In the case of a solitary pulmonary metastasis, surgical resection of the pulmonary metastasis leads to a cure in most instances. Giant cell tumor of bone is a locally aggressive tumor that is managed by surgery. Because it arises immediately adjacent to the articular cartilage, a resection requires removal of the articular surface. When the adjacent articular surface is unnecessary (e.g., proximal fibula), a resection is recommended. Otherwise, curettage is better. A simple curettage is insufficient and is followed by an unacceptable incidence of local recurrence (approximately 50%). A more aggressive curettage is associated with a lower incidence of recurrence, even though a recurrence rate of approximately 20% is considered acceptable. The simplest method for doing an “aggressive” curettage is to use a large window in the bone and burring the cavity with a high-speed burr. Phenol has been used to kill any residual cells and polymethyl methacrylate. Others have used cryosurgery, which reduces the incidence of recurrence lower than other methods but has its own potential complications. Resection of the lesion and autograft arthrodesis, allograft, and metallic implant have all been advocated in the past but now seem excessive, except when the deformity of the joint is so extreme as to require some sort of arthroplastic procedure to allow competent function. Lesions of bone sites that may be resected with minimal disability, such as the ilium or fibula, should be removed by surgical extirpation of the site. Most local recurrences can be treated with a second curettage with good results. There is no evidence that local recurrence leads to an increased risk in metastasis.

In this case, the patient presented late and thus the lesion had progressed beyond a point of conservative management. Limb salvage techniques like curettage cannot be used once there is vascular invasion and surrounding soft tissue destruction or there is superadded infection. Thus we conclude the importance of early diagnosis and management of giant cell tumors so that the limb can be saved.
